# Network pharmacology and molecular docking approaches predict the mechanisms of *Corididius chinensis* in treating manganese-induced nervous system diseases: A review

**DOI:** 10.1097/MD.0000000000035669

**Published:** 2023-10-27

**Authors:** Mei Zhang, Huixian Lou, Jing Ma, Keyi Xiong, Xiaohui Hou

**Affiliations:** a Zunyi Medical University, College of Basic Medicine, Zunyi, Guizhou, China

**Keywords:** *Corididius chinensis*, manganese-induced neurotoxicity, molecular docking, network pharmacology, neurodegenerative diseases

## Abstract

Neurotoxicity could be induced by long exposure to manganese (Mn). The traditional Chinese medicine, *Corididius chinensis* (Cc) has been proven to have a certain curative effect on Mn poisoning. Therefore, network pharmacology was performed to explore potential therapeutic targets and pharmacological mechanisms of Cc. We found ingredients by building our own database through literature, (which is the first to screen traditional Chinese medicine without traditional Chinese medicine systems pharmacology database and analysis platform databases and it is applicable whenever a Chinese medicine is not found in the traditional Chinese medicine systems pharmacology database and analysis platform database) and potential targets of Mn-induced nervous system diseases from the OMIM, GeneCards, and DrugBank database were identified. A protein-protein interaction network was constructed using Cytoscape. Gene ontology and Kyoto encyclopedia of genes and genomes pathway enrichment analysis was performed for the treatment of Mn-induced nervous system disease, and molecular docking was carried out to verify the results of network pharmacology analysis. After screening disease-related genes, 12 intersecting genes overlapped between 284 target proteins of the active compound and 195 potential disease targets. The pathways of neurodegeneration_multiple diseases and Alzheimer disease pathway may be the most potential pathway of Cc treating Mn-induced nervous system diseases. CASP9 and PTGS2 in neurodegeneration_multiple diseases, NOS1, NOS2 in Alzheimer disease pathway were identified as core targets. Especially, molecule docking analysis unveil that aspongpyrazine A docking NOS2 is the most potential therapeutic drug and target, which primarily involved in the processes of oxidative stress and inflammation.

## 1. Introduction

Fast-growing studies shown that neurodegenerative diseases are closely related to environmental transition metal exposure.^[1–3]^ Recent clinical trials have shown that long-term exposure to manganese (Mn) is associated with a high risk of developing neurodegenerative diseases, such as Parkinson disease (PD).^[4,5]^ Another study also demonstrated that PD model could be constructed reliably when rats inhaled Mn mixture.^[6]^ At the cellular level, Mn directly causes mitochondrial dysfunction by destroying mitochondrial complex I and consuming mitochondrial ATP storage. Moreover, the stress response in mitochondrion is activated, and ROS are stimulated to produce more toxic reactions and apoptosis.^[7]^ Clinically, the symptoms of Mn poisoning include depression, anxiety, cognitive impairment and autonomic dysfunction.^[8]^ Current treatment options for PD are mainly symptomatic because the etiological physiology has not been fully elucidated. Dopamine and other drugs have been shown to be effective in PD.^[9]^ However, more effective and less toxic medicines are expected to be developed to treat Mn-induced nervous system diseases.

Recently, traditional Chinese medicines (TCMs) have been considered for treating PD, with good tolerance.^[10]^ *Corididius chinensis* (Cc), a TCM, has been proven to have a certain curative effect on Mn poisoning.^[11,12]^ Moreover, the active biomolecules of Cc, which have been shown to have a protective function, were identified by GC–MS and other techniques. However, only proliferation in neural stem cells has been shown.^[13]^ Therefore, the underlying mechanism by which Cc treats Mn-induced nervous system diseases has not been fully verified.

Network pharmacology, which is based on bioinformatics and systems biology, is becoming increasingly dynamic. Network pharmacology is widely used in the in-depth study of TCM to explain scientific conundrums.^[14,15]^ The targets of the active compound of Cc and the disease targets of “Mn-induced neurotoxicity” and “Mn-induced nervous system disease” were collected to construct a component-target network and a PPI network of interaction targets. Afterwards, a gene ontology (GO) analysis was performed using the database for annotation, visualization, and integrated discovery (DAVID), followed by Kyoto encyclopedia of genes and genomes (KEGG) analysis.

**Figure 1.**
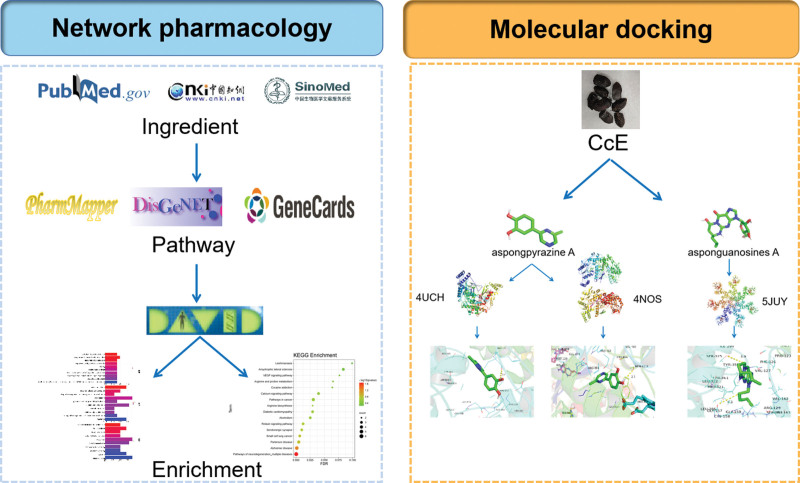
Schematic representation of the potential mechanism by which *Corididius chinensis* (Cc) protects against manganese (Mn)-induced neurotoxicity. The key ingredients and crucial components of Cc in treating Mn-induced neurotoxicity were filtered with network pharmacology; 2 previously shown bioactive compounds could bind tightly with 3 candidate molecular docking models, which was revealed after 48 molecular docking simulations.

We aimed to identify potential treatments for of Mn-induced nervous system diseases using network pharmacology for the first time, and to provide relevant relative references for subsequent research. The workflow is displayed in Figure 1.

## 2. Methods

### 2.1. Component collection

We obtained the main chemical components of Cc from China national knowledge infrastructure, universal databases, PubMed and Web of Science. The PubChem database (https://pubchem.ncbi.nlm.nih.gov/) was used to obtain certificate, transformation, sorting and classification information. The core bioactive components in Cc are asponguanosine A, aspongadenine B, asponguanine B, aspongpyrazine A, aspongpyrazine B, asponguanine C,^[13]^ N-acetyldopamine and 1/2-dehydro-N-acetyldopamine.^[16]^

### 2.2. Target prediction

We used ChemDraw 19.0 software to map the structure of the active components and predicted their targets using PharmMapper (http://lilab-ecust.cn/pharmmapper/index.html). The following filter criteria were applied: prediction accuracy (accuracy, AUC) > = 0. 7, predicted probability (probability, Prob) > 0. 9.^[17]^

Disease targets were then collected in the GeneCards database (http://www.genecards.org/), OMIM database (https://omim.org/), and Disgnet (https://www.disgenet.org/) with the keywords “Mn-induced the neurotoxicity and “Mn-induced nervous system disease.”^[18]^ A Venn diagram was drawn to integrate the common targets of Cc in nerve protection (https://bioinfogp.cnb.csic.es/tools/venny/).

### 2.3. Network construction

We constructed the “component-target” network using Cytoscape 3.7.0 software. The search tool for the retrieval of interacting genes proteins (STRING) (https://cn.string-db.org/cgi/input.pl) aims to integrate all publicly available sources of protein–protein interaction information and search for systems with known and predicted interactions between proteins.^[19]^ We collected targets using STRING and acquired the protein-protein interaction network (PPI) network. The data were imported into Cytoscape3. 7.0 software to obtain the PPI. The Network Analyser plug-in was then used to analyze the network topology parameters, and nodes with above-average nodules were screened to further construct a network of “top 10 targets network.” The Enrich database is commonly used for gene enrichment analysis. GO functional (cell function, molecular function, and biological function) analysis and KEGG pathway enrichment analysis of the coaction targets were conducted using the BINGO plugin and the DAVID database (https://david.ncifcrf.gov/).

### 2.4. Molecular docking

AutoDOCK is an open-source molecular docking software for systematic evaluation and activity screening, providing postanalysis tools for k-means and layered clustering methods based on the docking site (protein–ligand action) and compound properties (atomic composition), which can be directly introduced into ligand molecules for docking and allows multiple ligands to dock at the same time. The bioactive components and top 10 targets of Cc were selected for the molecular docking analysis. We downloaded the PDB format file of the 3D structure of the targets from the RCSB PDB (https://www.rcsb.org/) database and downloaded the mol2 format files of the 3D structure of the core active component from several other databases. Core ingredients were determined by ChemDraw 19.0. The software program minimizes the energy of the compound, uploads it in Mol format, and selects the standard binding mode from the server articles. The specific docking parameters are as follows: generic evolutionary method = 200, generation = 70, and number of solutions = 2.

## 3. Results

### 3.1. Data preparation

A total of 89 compounds in Cc were collected from several articles (see Supplementary Table 1, supplemental Content, http://links.lww.com/MD/K416, which demonstrates the compounds of Cc). A total of 284 potential targets of Cc-related targets and 195 targets of Mn-induced nervous system diseases were obtained. Duplicate data were removed, and 12 intersecting genes were identified as potential key targets of Cc in Mn-induced nervous system diseases.

### 3.2. Network construction

#### 3.2.1. Compound-target-pathway-disease network.

**Figure 2.**
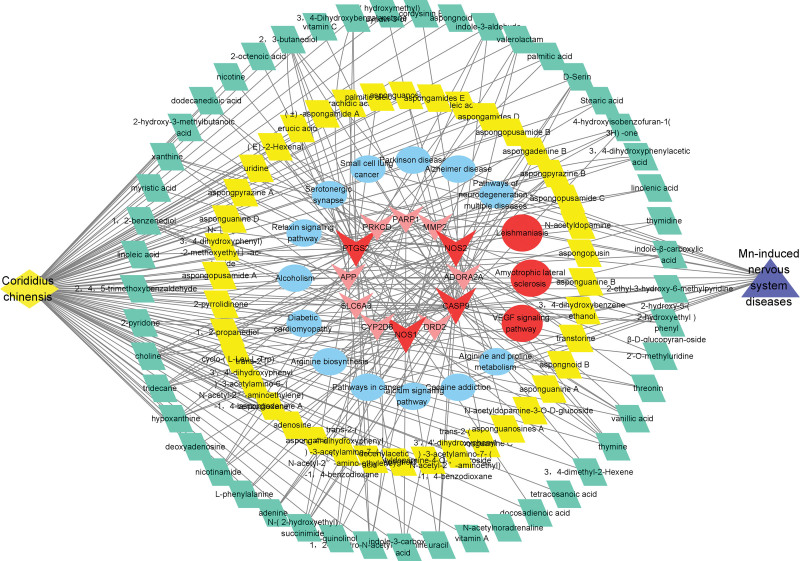
Interaction network of *Corididius chinensis* (Cc) components-targets-pathway-disease.

We established a 374-node network of the relationships among the 89 compounds and 284 targets using Cytoscape software (Fig. 2). The redder of the node in the network was positively related to its degree value. The corresponding targets of candidate ingredients were removed. Finally, 12 candidate pathways and 16 core targets were obtained from the network. After a comprehensive analysis, we concluded that the key targets are CASP9, PTGS2, NOS2 and NOS1.

#### 3.2.2. Venn diagram.

**Figure 3.**
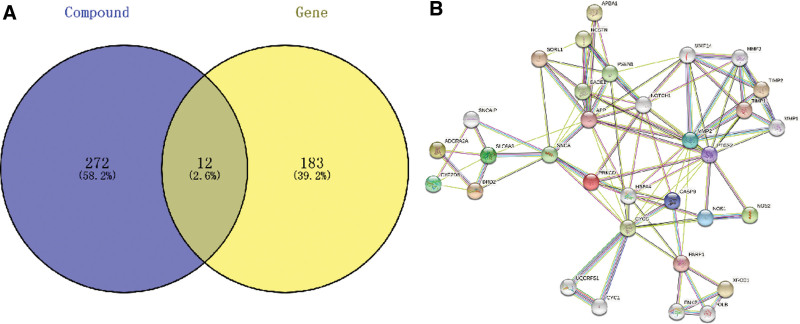
Venn diagram of the targets of *Corididius chinensis* and disease and PPI network for interacting targets. (A) Venn diagrams showing the number of potential targets of *Corididius chinensis* (Cc) in the context of manganese-induced neurotoxicity and the intersection of identified target genes of active compounds. (B) The figure shows the interacting targets in the PPI network.

We obtained 89 components of Cc from the China national knowledge infrastructure, PubMed and Web of Science databases. Twelve common targets intersected among the Cc components and Mn-induced nervous system diseases (Fig. 3A).

#### 3.2.3. PPI network construction.

The twelve targets were imported into the STRING database to construct the PPI network. Next, 12 nodes were obtained using this database, and the PPI network information from the STRING database was imported into Cytoscape software for visualization, revealing the top 12 core target networks (Fig. 3B). Consequently, 4 core proteins were identified, CASP9, PTGS2, NOS1, and NOS2, and individual molecular docking analyses were performed for each protein.

#### 3.2.4. GO and pathway enrichment analysis.

**Figure 4.**
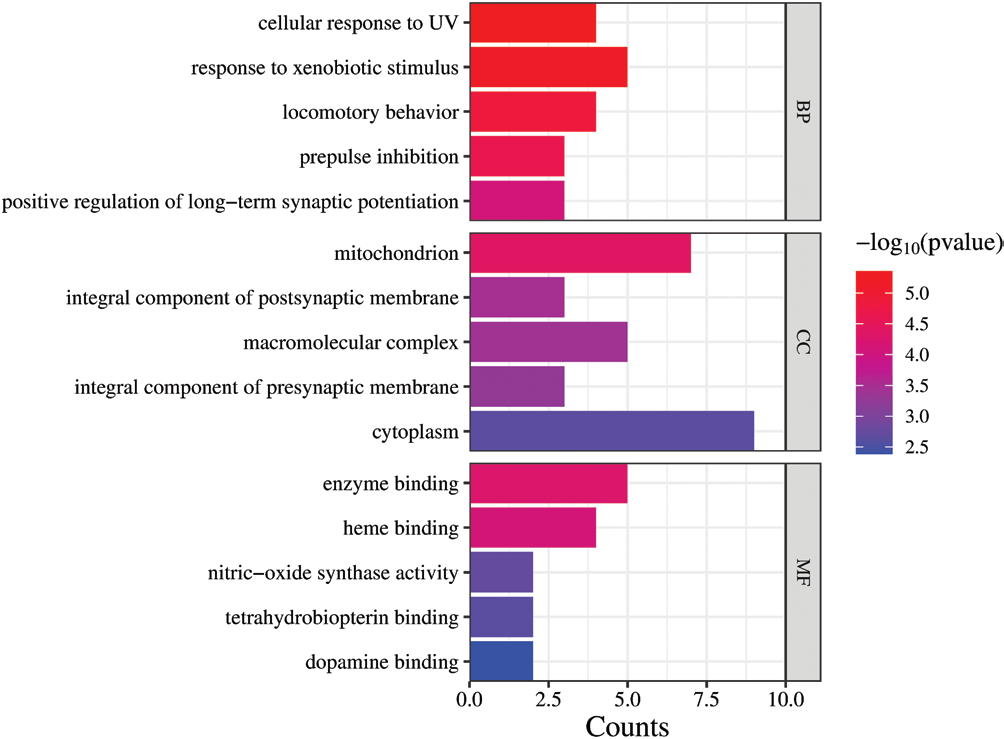
The top 10 results of all 3 groups (BP, CC, MF); enrichment analyses for 116 gene ontology (GO) items. The number of target genes in each pathway is represented by the x-axis, and the top 10 terms in the 3 groups (BP, CC, MF) are represented by the ordinate. Increasingly red colors indicate a lower *P* value. BP = biological process, CC = cellular component, Cc = *Corididius chinensis*, MF = molecular function.

The GO functional enrichment analysis of the top 10 targets was imported into the DAVID database, and 98 GO items were obtained, including 65 biological process (BP), 17 cellular component, and 16 molecular function processes. The top 5 items of BP, cellular component, molecular function were selected visually based on *P* values respectively (Fig. 4). The results showed that the effect of Cc was mainly related to BP, for example cellular response to UV, response to xenobiotic stimulus and locomotory behavior, prepulse inhibitios and positive regulation of long-term synaptic potentiation, indicating that the therapeutic effect of Cc against Mn-induced nervous system diseases was primarily associated with regulation of neural impulses. Among these 3 categories, the top significantly enriched terms indicated that the therapeutic effects of Cc in protecting against Mn-induced nervous system diseases may involve the cellular response to UV. These targets are involved in enzyme binding, heme binding, nitric-oxide synthase activity, and other biological functions. They play important roles in the mitochondrion, integral component of postsynaptic membrane and macromolecular complex.

**Figure 5.**
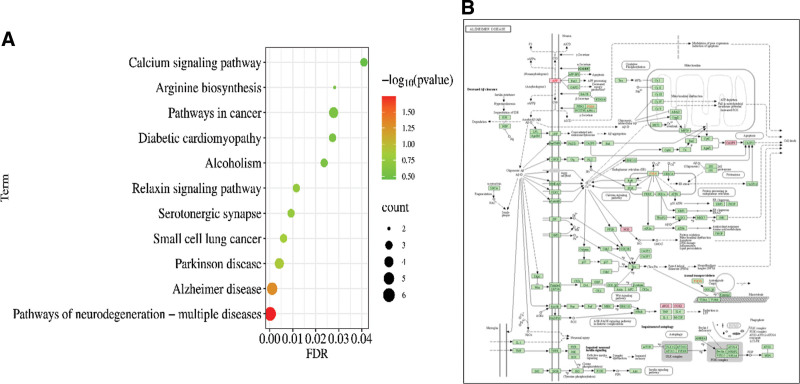
Pathways of CcE compound against manganese (Mn)-induced nervous system diseases (count > 1). (A) Bubble plot of top 16 Kyoto encyclopedia of genes and genomes (KEGG) pathways. (B) Alzheimer disease pathway downloaded from KEGG Database. Gene ratio = count/set size.

We conducted a KEGG pathway analysis of these target genes to better understand the underlying mechanisms of Cc in Mn-induced nervous system diseases. The top 11 pathways with statistically significant differences (*P* < .05) were identified using KEGG analysis (Fig. 5A). Regarding drug effects, pathways of neurodegeneration_multiple diseases Alzheimer disease pathway, PD and small cell lung cancer pathway were the potential pathways. The KEGG combined with GO analysis results and screened key targets suggested that Cc played a role against Mn-induced nervous system diseases mainly via the pathways of neurodegeneration_multiple diseases (Fig. 5B) and Alzheimer disease pathway.

### 3.3. Molecular docking

**Figure 6.**
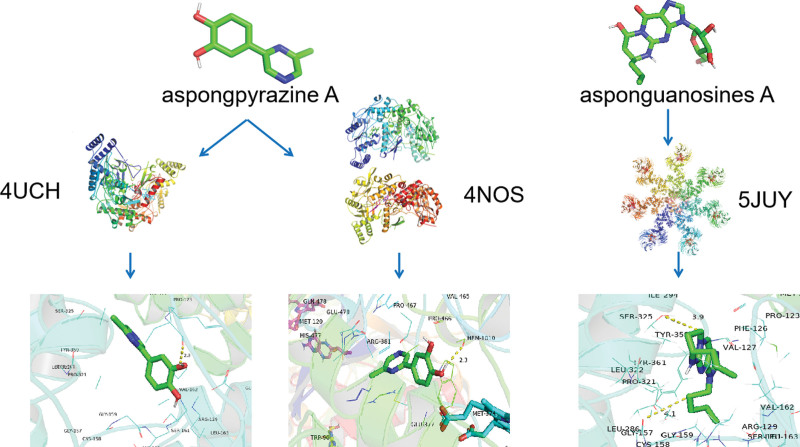
Molecular 3D models of bioactive compounds binding to their predicted protein targets. Green lines in the respective binding sites represent residues. Hydrogen bonds were represented by yellow dashed lines. The distances of interaction are indicated around the site of bonding. 3D interaction diagrams of aspongpyrazine A in the active site of 4HB (PDB ID 4UCH). 3D interaction diagrams of aspongpyrazine A in the active site of 4HB (PDB ID 4NOS). 3D interaction diagrams of asponguanosines A in the active site of DTP (PDB ID 5JUY).

We optimized and processed the binding patterns of the target proteins and their components in PyMoL 2.5.4. There are strong interactions between the 2 molecules, including electrostatic interaction, Van der Waals force, hydrogen bond, hydrophobic interaction, etc. This energy is expressed as binding energy. In molecular docking, a lower the affinity standard indicates a stronger binding affinity. The results showed that most affinity values are below −5 kcal/mol, indicating that the core targets have a relatively stable binding energy with the active components.^[12]^ We selected the top 4 target proteins (CASP9, PTGS2, NOS1, and NOS2) in the PPI network with a degree > −6 in the “compound-target-pathway-disease” network. The primary active components (including asponguanosines A, aspongadenine B, asponguanine B, aspongpyrazine A, aspongpyrazine B, and asponguanine C) were molecularly docked by AutoDock Vina, as shown in Table 1. As shown, asponguanosine A, aspongadenine B, asponguanine B, aspongpyrazine A, aspongpyrazine B, and asponguanine C all docked tightly with NOS2. The molecular binding energy of 18 pairs exceeded 5 kcal/mol, indicating a strong ability of Cc bioactive components to bind Mn-induced nervous system disease-related proteins. NOS2 demonstrated the strongest affinity for aspongpyrazine A (Table 1). The integrated analysis revealed that the docking scores of asponguanosine A with NOS1 were poor. Thus, aspongpyrazine A could be the most promising molecule for treating Mn-induced nervous system diseases among the compounds tested (Fig. 6).

**Table 1:** Docking results of core target proteins with bioactive components.

Compound\Afffinity(kcal/mol)	CASP9 (5JUY-DTP)	PTGS2 (5IKQ-JMS)	NOS1 (4UCH-4HB)	NOS2 (4NOS-H4B)
Asponguanosines A	−7.4	−5.4	4.7	−6.4
Aspongadenine B	−5.7	−7	−4.7	−6.5
Asponguanine B		−5.8	−5	−6.7
Aspongpyrazine A	−6.4	−4.9	−8	−8.1
Aspongpyrazine B	−4.7		−5.3	−5.6
Asponguanine C	−5.9	−7.2	−5.2	−6.6

## 4. Discussion

In this research, the potential targets and material foundation of Cc for treating Mn-induced nervous system diseases were analyzed utilizing network pharmacology. Eighty-nine components were collected from several articles, which have good biological activities. Some of the screened compounds have good biological activities. The structure of the active ingredients was determined in ChemDraw 3D, and the results were input into the PharmMapper database for target screening. The disease targets of “Mn-induced neurotoxicity” and “Mn-induced nervous system disease” were collected from the DisGeNET, OMIM and GeneCards databases. Mn directly causes mitochondrial dysfunction by destroying mitochondrial complex I and consuming stored mitochondrial ATP. Thus, the stress response in mitochondria is activated, and ROS are stimulated to produce more toxic reactions and apoptosis,^[7]^ potentially leading to neurodegenerative disease.^[8]^ Neurotoxicity belongs to the category of “tremor syndrome” in TCM. Tremor syndromes originate in the brain. Old age and low immunity are the causes, while renal insufficiency is the root. In TCM, the renal insufficiency is closely related to the decline of reproductive ability. The Compendium of Materia Medica records that Cc is mainly used to adjust body balance and renal insufficiency. Therefore, from the perspective of TCM, Cc is suitable for toxic neurodegenerative diseases patients. Modern pharmacological studies have demonstrated that Cc has a certain curative effect against Mn poisoning. Cc reduced the number of apoptotic cells and the expression of caspase-3, cytochrome c, and malondialdehyde, improving the reproductive system compared with Mn poisoning group, because its antioxidant activity.^[11]^ The blood-testis barrier, including boundary adhesion molecule 1, zonal occlusion-1, claudin1 and occludin were ameliorated by Cc in Mn poisoning.^[12]^ Asponguanine B in Cc have significantly neuroprotective effects, but only the proliferation stimulating properties of neural stem cells.^[9]^ Therefore, we speculate that Cc plays a therapeutic role in Mn-induced nervous system diseases.

Our network analysis demonstrated that the most highly correlated targets between Mn-induced nervous system disease-related genes and Cc compound-related genes were CASP9, PTGS2, NOS1 and NOS2, which are primarily involved in oxidative stress and inflammation. The results of PPI network identified CASP9, PTGS2, NOS1, and NOS2. In particular, CASP9 may be the core target.

In wild-type neurons, cell death occurs through the caspase-9 pathway, which relies on the key molecule CASP9. The existence of these 2 alternative pathways of nutrient deprivation induced death may have an impact on ensuring the correct development of the nervous system. In wild-type neurons, death requires CASP2 dependent pathway, and caspase-9 dependent pathway seems to be inhibited by endogenous inhibitor of apoptosis protein. On the contrary, for CASP2-null neurons, death depends on the CASP9 pathway.^[20,21]^ Therefore, the anti-Mn-induced nervous system diseases potential of CASP9 still deserves further attention and research, and CASP9 may be an important target for the treatment of Mn-induced nervous system diseases.

PTGS2 is an important inflammatory mediator mostly expressed in the central nervous system; high PTGS2 expression is associated with neuronal damage and cognitive impairment. Multiple single-nucleotide polymorphism have been described in the promoter region of PTGS2 gene, which may regulate its transcription, but only one polymorphism is located at position 765 G/C, and the stimulator-1 binding site inferred by a has been proven to be functional.^[22]^ PTGS2 polymorphism is associated with reduced risk of Alzheimer disease.^[22,23]^ Michele et al reported that the polymorphism of the PTGS2 gene is associated with an unrelated incidence of stent restenosis.^[24]^ Nevertheless, the potential of PTGS2 for atherosclerosis still deserves further attention. PTGS2 as an important substance that affects inflammation is likely to become a key target for the treatment of Mn-induced nervous system diseases.

Schwann cells are essential in the peripheral nervous system. The dysfunction of Schwann cells can induce various degenerative disease of peripheral nerves. Oxidative stress is considered a pathogenic factor of degenerative neurological diseases. However, in peripheral nerve degenerative disease, there is no effective molecule that can be used to inhibit the neurodegeneration of nerve Degenerative disease. Ethyl pyruvate acid is a candidate regulator of oxidative stress, targeting Schwann cells during peripheral nerve degeneration. Some studies have showen that inhibiting NOS1 can prevent neuronal degeneration.^[25,26]^ Another study demonstrated that deleting the NOS2 gene protects against Mn-induced neurological dysfunction in mice.^[27]^ As important substances that affect oxidative stress, NOS1 and NOS2 are an important factors in the occurrence and development of Mn-induced nervous system diseases.

In GO functional enrichment analysis, Cc for the treatment of Mn-induced nervous system diseases is mainly the mitochondrion, while the molecular function mainly affects enzyme binding and the biological process mainly affects cellular response to UV, response to xenobiotic stimulus. These findings apply to the results of the current research. It has been reported that Cc increases the total antioxidant capacity, and glutathione peroxidase enzymes in serum of Mn-exposed rats with high dose of Cc treatment, which increased the ability of rats to resist external stimuli.^[11]^ In KEGG pathway enrichment analysis, pathways of neurodegeneration_multiple diseases and Alzheimer disease may be the main pathways for Cc treatment of Mn-induced nervous system diseases. Pathways of neurodegeneration_multiple diseases are the top-ranked signaling pathways, and CASP9, APP, NOS2, NOS1, PTGS2 and SLC6A3 are all intersecting targets enriched in this pathway. Among them, CASP9, PTGS2, NOS1 and NOS2 are key targets identified from the network analysis. The Alzheimer disease pathway is the second-ranked signaling pathway, and 5 key targets, CASP9, APP, NOS2, NOS1 and PTGS2, are enriched in this pathway. They cover the entire range of calcium signaling in this process and play an integral and important role in AGE-RAGE signaling in this pathway.^[28]^ After predicting and analyzing potential targets and pathways, we surmised that the effect of Cc in ameliorating Mn-induced nervous system diseases involves inhibiting the inflammatory response and oxidative stress.

In molecular docking, Cc has a high probability of binding to 4 key targets CASP9, PTGS2, NOS1, and NOS2. According to previous studies, the active components in Cc, including asponguanosines A, aspongadenine B, asponguanine B, aspongpyrazine A, aspongpyrazine B, and asponguanine C, are not clearly understood and have not been characterized in animal models. Among them, asponguanine B exerts better neuroprotective effects, but only in stimulating the proliferation of neural stem cells.^[9]^ These findings suggest that these ingredients may be critical in the treatment of Mn-induced nervous system diseases, which is worthy of further discussion. The docking results showed that the 2 compounds, asponguanosines A and aspongpyrazine A, could strongly bind to the proteins, and the binding of aspongpyrazine A to NOS2 showed the lowest binding energy, indicating that the combination was the most stable. However, the effect of aspongpyrazine A on NOS2 has not been reported in the literature and needs to be confirmed by relevant research.

Although this study predicted the potential targets and mechanisms of Cc in the treatment of Mn-induced nervous system diseases, it still had some limitations. We used only network pharmacology and molecular docking methods, which are modern bioinformatics methods, to explore the role of Cc in Mn-induced nervous system diseases. However, the current network information technology needs to be further improved, and the accuracy and timeliness of database data require scientific verification. The influence and mechanisms of these potential active ingredients on Mn-induced nervous system diseases have not been explained and verified; however, it is believed that they exhibit substantial potential for development and research.

## 5. Conclusion

This study predicts the potential targets and mechanisms of action of Cc in the treatment of Mn-induced nervous system diseases. Cc can treat Mn-induced nervous system diseases through targets such as CASP9, PTGS2, NOS1, and NOS2. Its mechanism of action may be related to pathways of neurodegeneration_multiple diseases and Alzheimer disease pathway, which is valuable for further research. Nevertheless, our research lacks experimental validation. The binding of aspongpyrazine A to NOS2 has a good binding energy of − 8.1 kcal/mol. Future experimental studies are expected to be conducted, confirming our findings and continuing to explore new drugs for treating Mn-induced nervous system diseases on this basis. This study is the first to screen the TCM without traditional Chinese medicine systems pharmacology database and analysis platform databases and it is applicable at any time. Building one own TCM database is beneficial for ensuring the integrity of ingredients and targets, which means that some of the latest bioactive molecules that have not yet been included in the traditional Chinese medicine systems pharmacology database and analysis platform database are included in our database.

## Author contributions

**Conceptualization:** Huixian Lou, Jing Ma, Xiaohui Hou.

**Data curation:** Mei Zhang, Huixian Lou, Keyi Xiong, Xiaohui Hou.

**Formal analysis:** Mei Zhang, Jing Ma.

**Funding acquisition:** Xiaohui Hou.

**Methodology:** Mei Zhang, Huixian Lou, Keyi Xiong.

**Resources:** Mei Zhang, Huixian Lou.

**Software:** Mei Zhang, Jing Ma.

**Validation:** Keyi Xiong.

**Visualization:** Mei Zhang, Huixian Lou, Keyi Xiong.

**Writing – original draft:** Mei Zhang.

**Writing – review & editing:** Mei Zhang, Xiaohui Hou.

## Supplementary Material


