# Supplementary material for: Network pharmacology and molecular docking approaches predict the mechanisms of Corididius chinensis in treating manganese-induced nervous system diseases: A review
Source: Medicine (Baltimore). 2023 Oct 27;102(43):e35669. doi: 10.1097/MD.0000000000035669 (PMC10615487; doi:10.1097/MD.0000000000035669)
Supplement: Supplementary file 1 [file medi-102-e35669-s001.docx]

| **STable 1.** Eighty-nine components of Corididius chinensis classification. | **No** | **Compounds** | **molecular formula** | **Structure** | **OB(%)** | **DL** | **MOLID** |
| --- | --- | --- | --- | --- | --- | --- | --- |
| Nucleotides | N1 | asponguanine A | C_11_H_14_N_4_O_3_ | 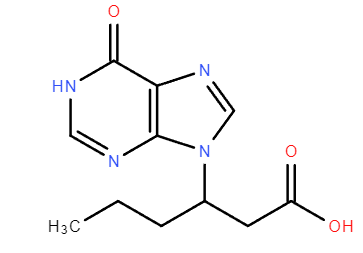 | _ | _ | _ |
|  | N2 | asponguanine B | C_11_H_16_N_4_O_2_ | 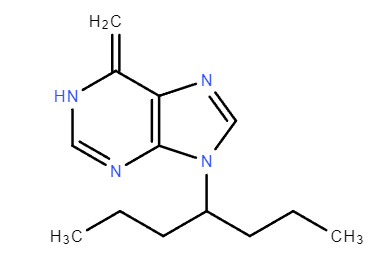 | _ | _ | _ |
|  | N3 | thymine | C_5_H_6_N_2_O_2_ | 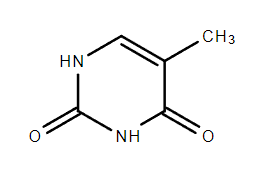 | 74.2 | 0.02 | MOL006953 |
|  | N4 | thymidine | C_10_H_14_N_2_O_5_ | 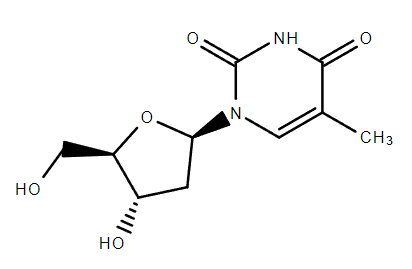 | 11.34 | 0.11 | MOL006240 |
|  | N5 | uracil | C_4_H_4_N_2_O_2_ | 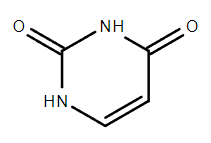 | 42.53 | 0.02 | MOL001744 |
|  | N6 | adenine | C_5_H_5_N_5_ | 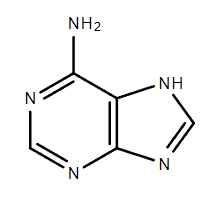 | 62.81 | 0.03 | MOL001788 |
|  | N7 | hypoxanthine | C_5_H_4_N_4_O | 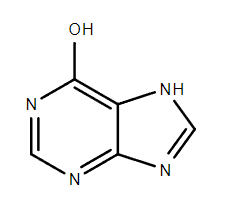 | 52.29 | 0.04 | MOL001831 |
|  | N8 | xanthine | C_5_H_4_N_4_O_2_ | 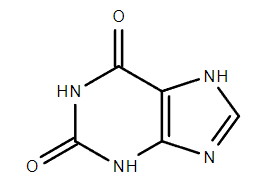 | 46.17 | 0.04 | MOL010716 |
|  | N9 | asponguanine C | C_9_H_10_N_4_O_3_ | 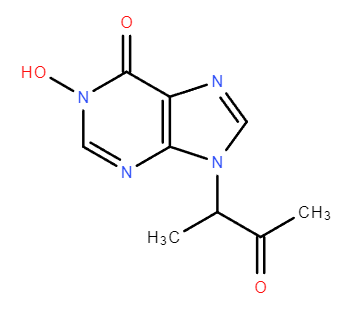 | _ | _ | _ |
|  | N10 | asponguanine D | C_10_H_12_N_4_O_2_ | 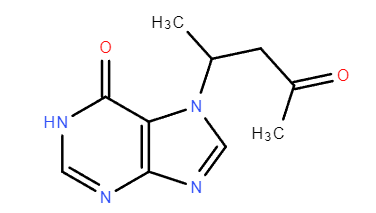 | _ | _ | _ |
|  | N11 | aspongadenine A | C_12_H_17_N5O_2_ | 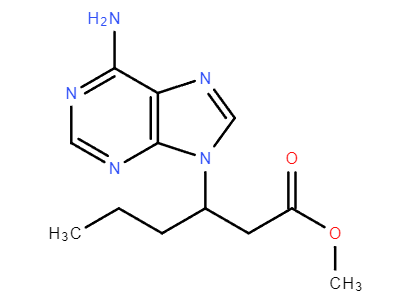 | _ | _ | _ |
| Nucleotides | N12 | aspongadenine B | C_12_H_17_N5O | 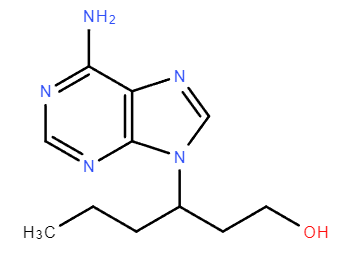 | _ | _ | _ |
|  | N13 | 2'-O-methyluridine | C_10_H_14_N_2_O_6_ | 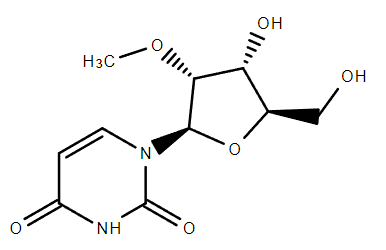 | _ | _ | _ |
|  | N14 | deoxyadenosine | C_10_H_13_N_5_O_3_ | 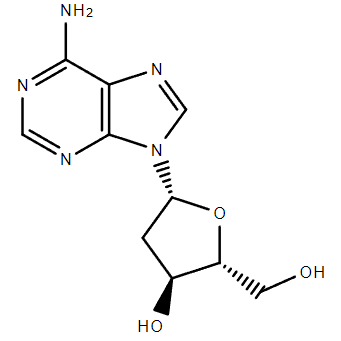 | 30.13 | 0.15 | MOL009000 |
|  | N15 | adenosine | C_10_H_13_N_5_O_4_ | 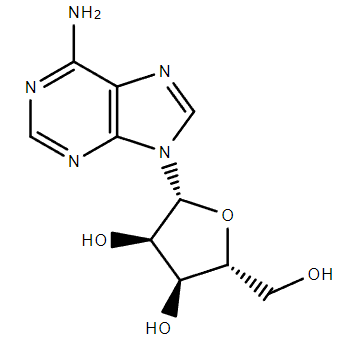 | 15.98 | 0.18 | MOL001787 |
|  | N16 | cordysinin B | C_11_H_15_N_5_O_4_ | 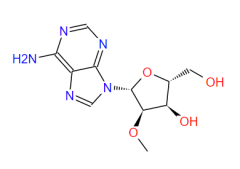 | _ | _ | _ |
|  | N17 | uridine | C_9_H_12_N_2_O_6_ | 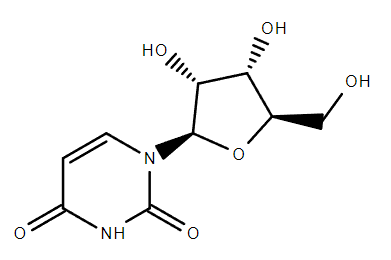 | 10.49 | 0.11 | MOL000059 |
|  | N18 | asponguanosines A | C_16_H_23_N_5_O_6_ | 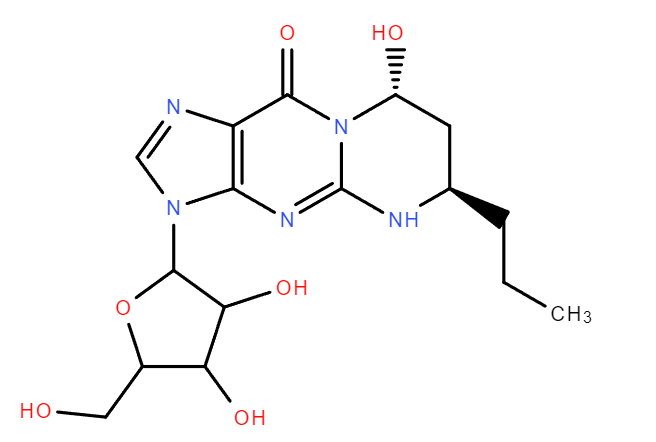 | _ | _ | _ |
|  | N19 | asponguanosines B | C_16_H_23_N_5_O_6_ | 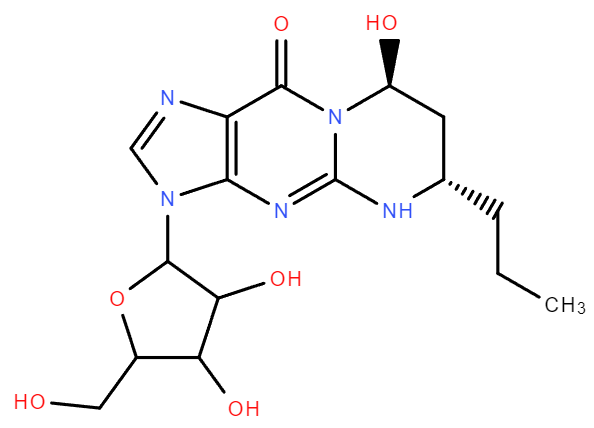 | _ | _ | _ |
| Nucleotides | N12 | aspongadenine B | C_12_H_17_N5O | 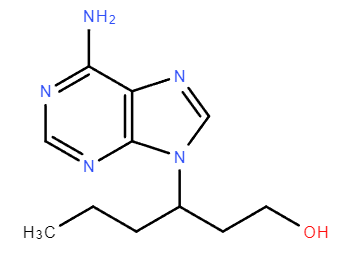 | _ | _ | _ |
|  | N13 | 2'-O-methyluridine | C_10_H_14_N_2_O_6_ | 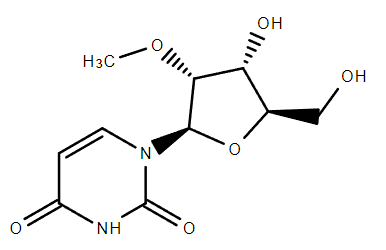 | _ | _ | _ |
|  | N14 | deoxyadenosine | C_10_H_13_N_5_O_3_ | 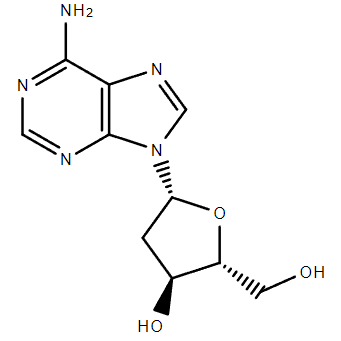 | 30.13 | 0.15 | MOL009000 |
|  | N15 | adenosine | C_10_H_13_N_5_O_4_ | 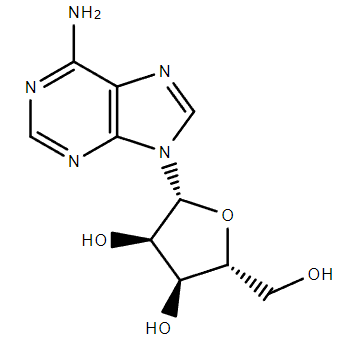 | 15.98 | 0.18 | MOL001787 |
|  | N16 | cordysinin B | C_11_H_15_N_5_O_4_ | 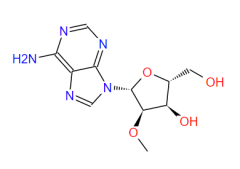 | _ | _ | _ |
|  | N17 | uridine | C_9_H_12_N_2_O_6_ | 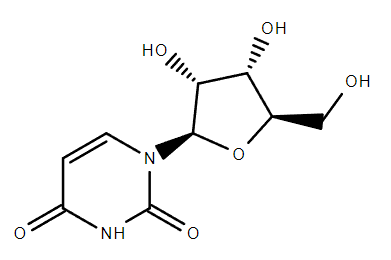 | 10.49 | 0.11 | MOL000059 |
|  | N18 | asponguanosines A | C_16_H_23_N_5_O_6_ | 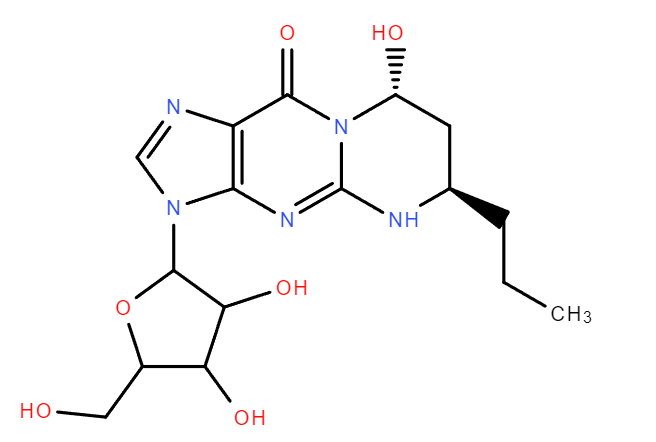 | _ | _ | _ |
|  | N19 | asponguanosines B | C_16_H_23_N_5_O_6_ | 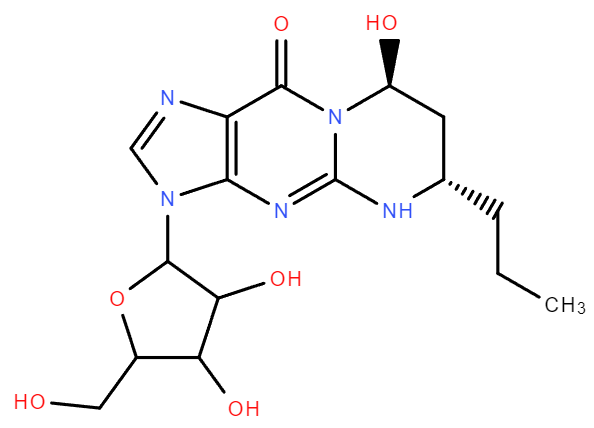 | _ | _ | _ |
| Dopamine analogues | D1 | 1，2-dehydro-N-acetyldopamine | C_10_H_11_NO_3_ | 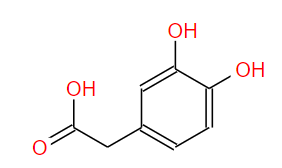 | _ | _ | _ |
|  | D2 | L-phenylalanine | C_9_H_11_NO_2_ | 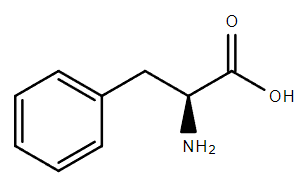 | 41.62 | 0.04 | MOL000041 |
|  | D3 | aspongamides D | C_11_H_15_NO_5_ | 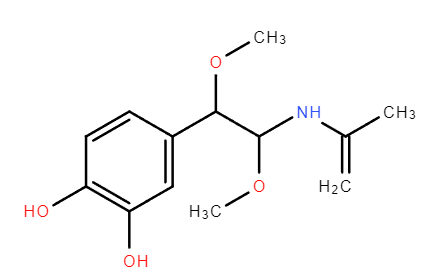 | _ | _ | _ |
|  | D4 | aspongamides E | C_12_H_17_NO_5_ | 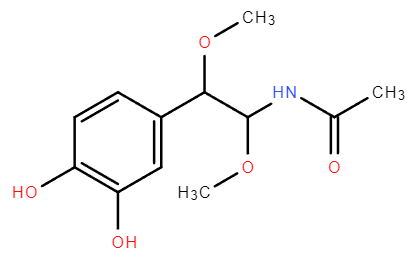 | _ | _ | _ |
|  | D5 | aspongamides C | C_17_H_18_N_2_O_5_ | 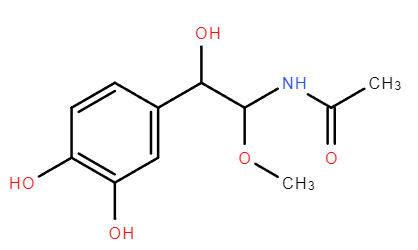 | _ | _ | _ |
|  | D6 | ( ±) -aspongamide A | C_30_H_31_N_3_O_9_ | 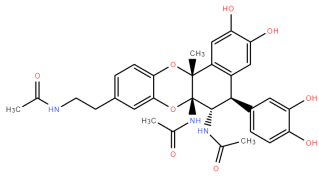 | _ | _ | _ |
|  | D7 | aspongopusamide A | C_20_H_20_N_2_O_6_ | 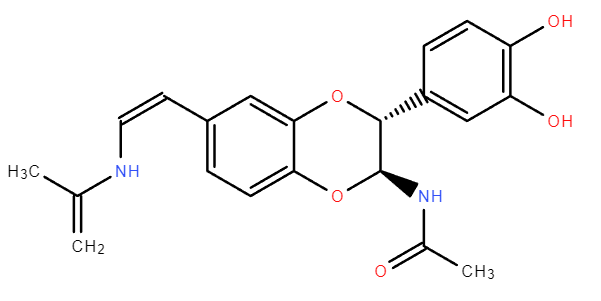 | _ | _ | _ |
|  | D8 | N-acetyldopamine-3-O-D-glucoside | C_16_H_23_NO_8_ | 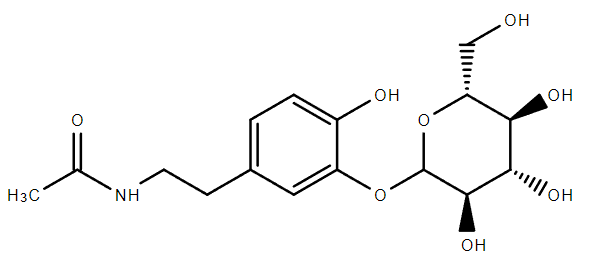 | _ | _ | _ |
|  | D9 | N-acetyldopamine-4-O-D-glucoside | C_10_H_23_NO_8_ | 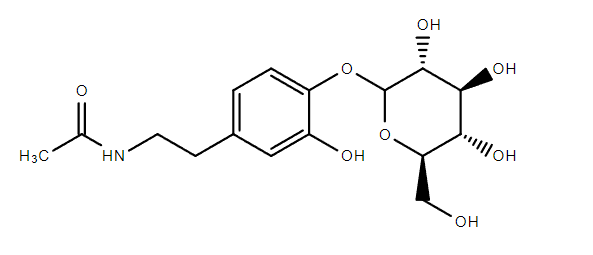 | _ | _ | _ |
|  | D10 | N-acetyldopamine | C_10_H_13_NO_3_ | 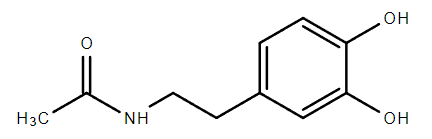 | _ | _ | _ |
| Dopamine analogues | D11 | N-acetylnoradrenaline | C_10_H_13_NO_4_ | 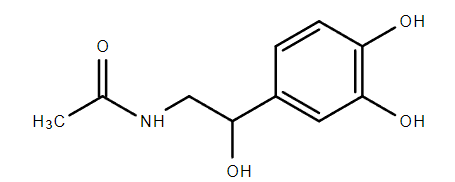 | _ | _ | _ |
|  | D12 | N-［2-( 3，4-dihydroxyphenyl) -2-methoxyethyl］-ac- etamide | C_11_H_15_NO_4_ | 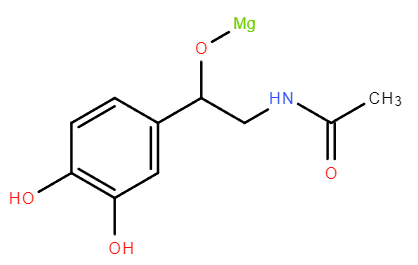 | _ | _ | _ |
|  | D13 | trans-2-( 3'，4'-dihydroxyphenyl ) -3-acetylamino-6- ( N-acetyl-2″-aminoethylene) -1，4-benzodioxane | C_20_H_20_N_2_O_6_ | 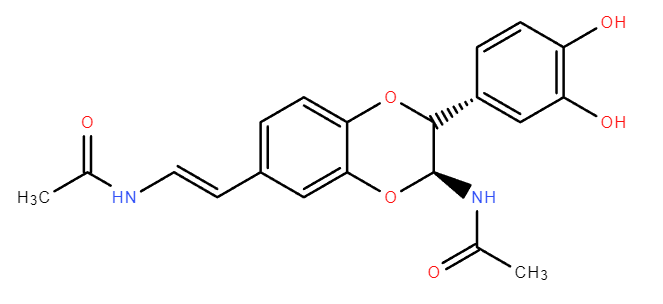 | _ | _ | _ |
|  | D14 | trans-2-( 3'，4'-dihydroxyphenyl ) -3-acetylamino-7- ( N-acetyl-2″-amino-ethylene) -1，4-benzodioxane | C_20_H_22_N_2_O_6_ | 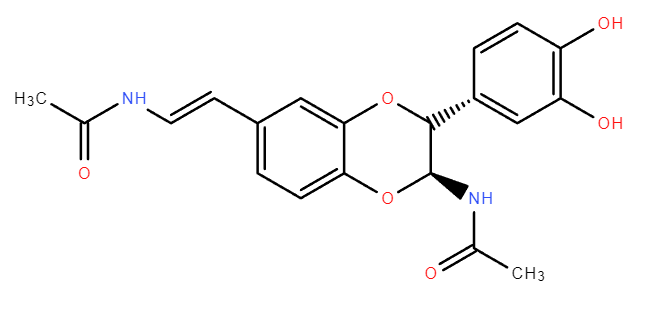 | _ | _ | _ |
|  | D15 | trans-2-( 3'，4'-dihydroxyphenyl ) -3-acetylamino-7- ( N-acetyl-2″-aminoethyl) -1，4-benzodioxane | C_20_H_22_N_2_O_6_ | 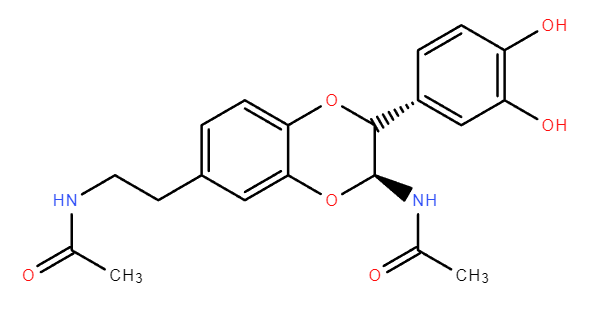 | _ | _ | _ |
|  | D16 | aspongopusamide B | C_20_H_20_N_2_O_6_ | 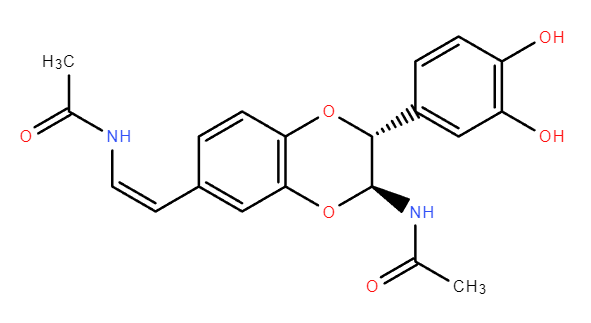 | _ | _ | _ |
| Dopamine analogues | D17 | aspongopusamide C | C_13_H_19_NO_6_ | 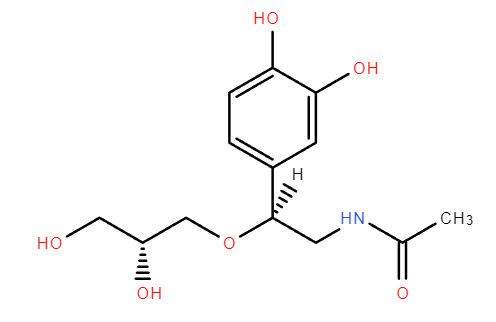 | _ | _ | _ |
| Nutritional ingredient | Y1 | oleic acid | C_18_H_34_O_2_ | 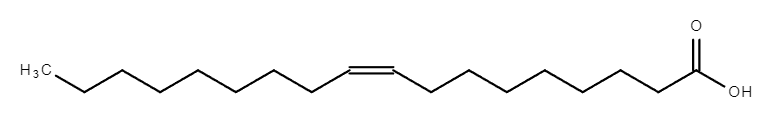 | 33.13 | 0.14 | MOL000675 |
|  | Y2 | linoleic acid | C_18_H_32_O_2_ | 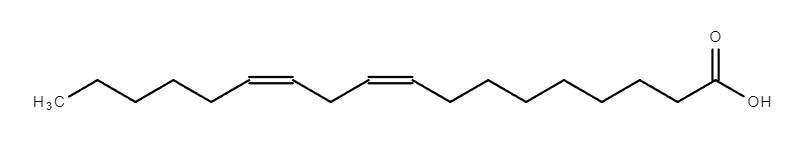 | 41.9 | 0.14 | MOL000131 |
|  | Y3 | palmitic acid | C_16_H_32_O_2_ | 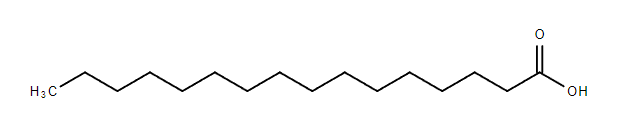 | 19.3 | 0.1 | MOL000069 |
|  | Y4 | Stearic acid | C_18_H_36_O_2_ | 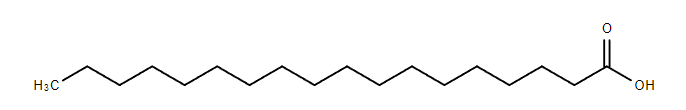 | 17.83 | 0.14 | MOL000860 |
|  | Y5 | linolenic acid | C_18_H_30_O_2_ | 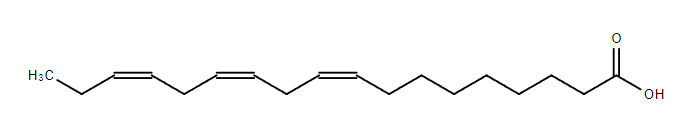 | 45.01 | 0.15 | MOL005500 |
|  | Y6 | myristic acid | C_14_H_28_O_2_ | 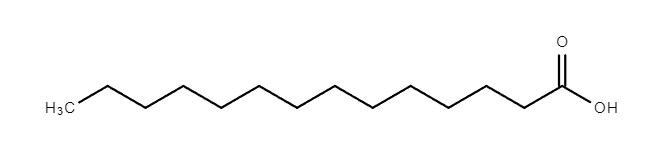 | 21.18 | 0.07 | MOL001393 |
|  | Y7 | dodecanedioic acid | C_22_H_44_O_2_ | 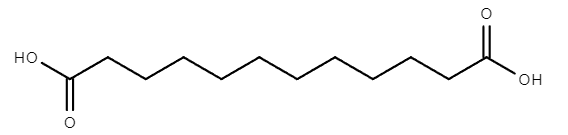 | _ | _ | _ |
|  | Y8 | docosadienoic acid | C_22_H_40_O_2_ | 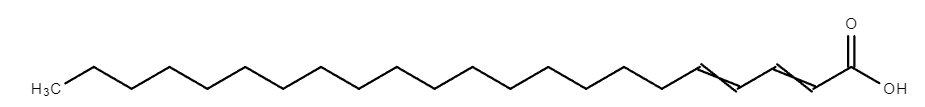 | _ | _ | _ |
|  | Y9 | tetracosanoic acid | C_24_H_48_O_2_ | 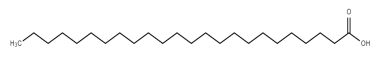 | 14.9 | 0.33 | MOL000663 |
|  | Y10 | Palmitoleic Acid | C_16_H_30_O_2_ | 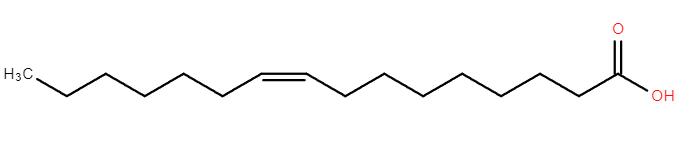 | 35.78 | 0.1 | MOL001739 |
|  | Y11 | arachidic acid | C_20_H_40_O_2_ | 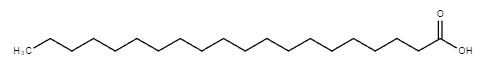 | 16.66 | 0.19 | MOL000012 |
|  | Y12 | erucic acid | C_13_H_24_O_2_ | 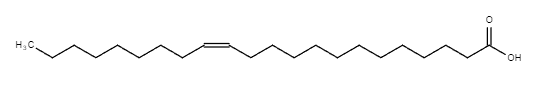 | 28.56 | 0.26 | MOL001631 |
|  | Y13 | vitamin A | C_20_H_30_O | 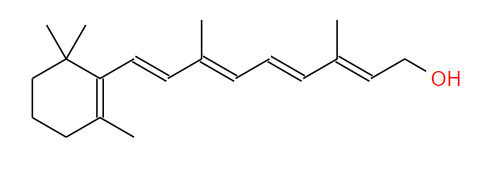 | 19.53 | 0.16 | MOL005567 |
|  | Y14 | vitamin C | C_6_H_8_O_6_ | 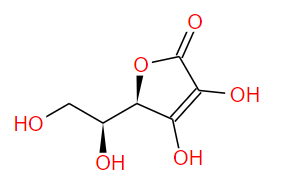 | 13.34 | 0.37 | MOL001691 |
| Nutritional ingredient | Y15 | D-Serin | C_3_H_7_NO_3_ | 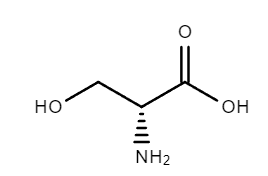 | 83.59 | 0.01 | MOL000064 |
|  | Y16 | threonin | C_4_H_9_NO_3_ | 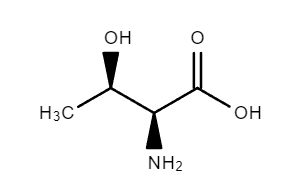 | 73.52 | 0.01 | MOL003971 |
| Aromatic compounds | A1 | tridecane | C_13_H_28_ | 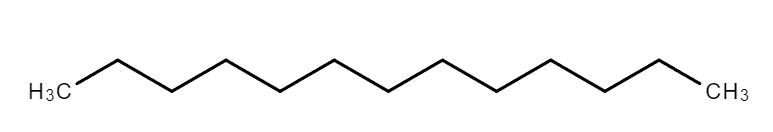 | 17.89 | 0.03 | MOL000610 |
|  | A2 | ( E) -2-Hexenal | C_6_H_10_O | 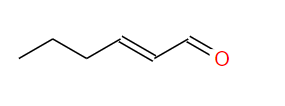 | 46.01 | 0.01 | MOL002675 |
|  | A3 | trans-2-decenylacetic acid | C_12_H_22_O_2_ | 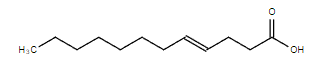 | _ | _ | _ |
|  | A4 | 3，4-dimethyl-2-Hexene | C_8_H_16_ | 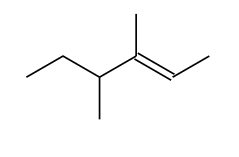 | _ | _ | _ |
|  | A5 | 2-octenoic acid | C_8_H_14_O_2_ | 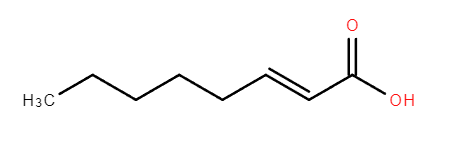 | 43.49 | 0.02 | MOL004592 |
| Other ingredients | O1 | choline | C_5_H_14_NO^+^ | 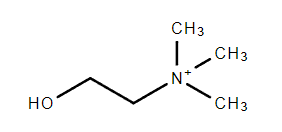 | 0.47 | 0.01 | MOL000394 |
|  | O2 | 2-pyrrolidinone | C_4_H_7_NO | 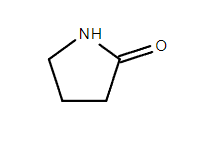 | 15.27 | 0.01 | MOL007547 |
|  | O3 | 2-ethyl-3-hydroxy-6-methylpyridine | C_8_H_11_NO | 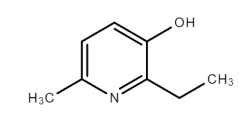 | _ | _ | _ |
|  | O4 | valerolactam | C_5_H_9_NO | 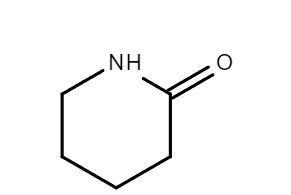 | _ | _ | _ |
| Other ingredients | O5 | 2-pyridone | C_5_H_5_NO | 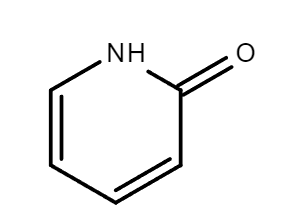 | _ | _ | _ |
|  | O6 | nicotinamide | C_6_H_6_N_2_O | 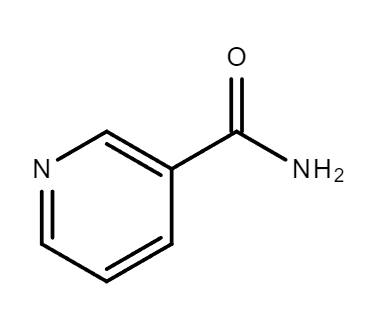 | 71.13 | 0.02 | MOL000857 |
|  | O7 | nicotine | C_10_H_14_N_2_ | 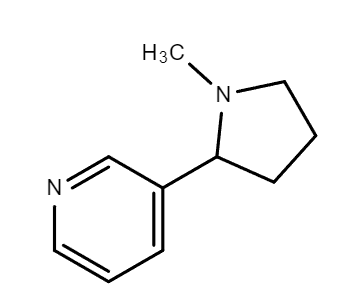 | 77.67 | 0.04 | MOL003403 |
|  | O8 | 2-quinolinol | C_9_H_7_NO | 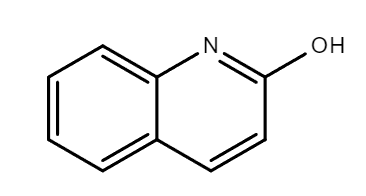 | _ | _ | _ |
|  | O9 | 6-( hydroxymethyl) pyridin-3-ol | C_6_H_7_NO_2_ | 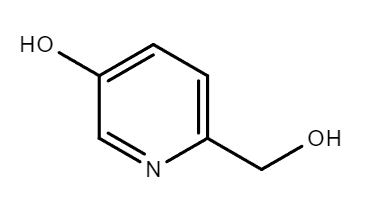 | _ | _ | _ |
|  | O10 | aspongpyrazine B | C_7_H_10_N_2_O_2_ | 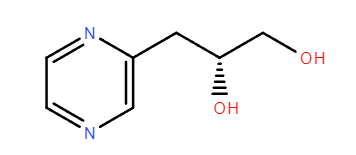 | _ | _ | _ |
|  | O11 | transtorine | C_10_H_7_NO_3_ | 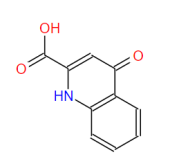 | 44.72 | 0.08 | MOL010768 |
|  | O12 | aspongpyrazine A | C_11_H_10_N_2_O_2_ | 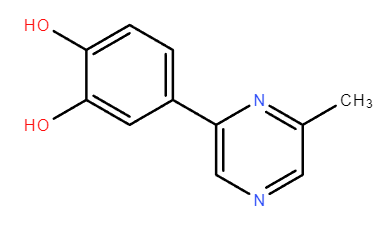 | _ | _ | _ |
|  | O13 | N-( 2-hydroxyethyl) succinimide | C_6_H_9_NO_3_ | 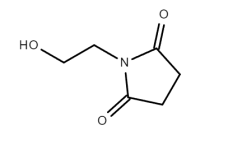 | _ | _ | _ |
|  | O14 | indole-β-carboxylic acid | C_9_H_7_NO_2_ | 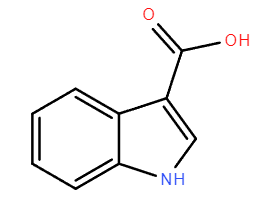 | _ | _ | _ |
| Other ingredients | O15 | indole-3-aldehyde | C_9_H_7_NO | 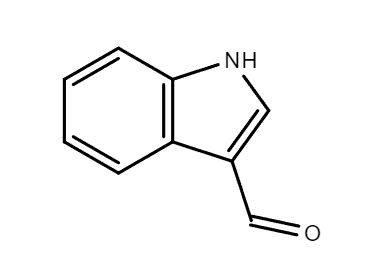 | 19.82 | 0.04 | MOL001762 |
|  | O16 | indole-3-carboxylic acid | C_9_H_7_NO_2_ | 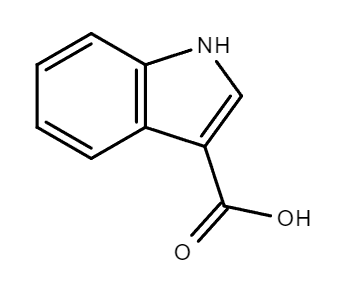 | 33.86 | 0.05 | MOL001737 |
|  | O17 | aspongnoid A | C_21_H_30_O_9_ | 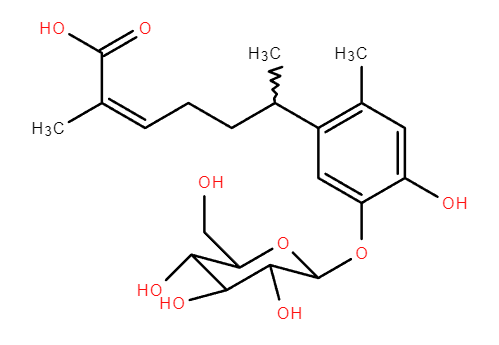 | _ | _ | _ |
|  | O18 | aspongnoid B | C_21_H_30_O_9_ | 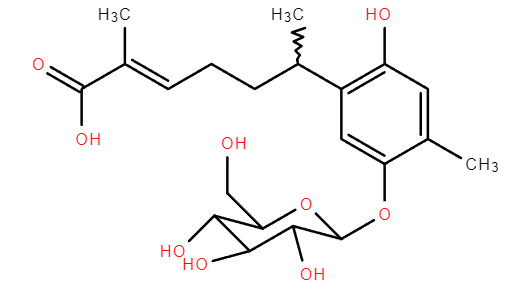 | _ | _ | _ |
|  | O19 | aspongnoid C | C_15_H_24_O_5_ | 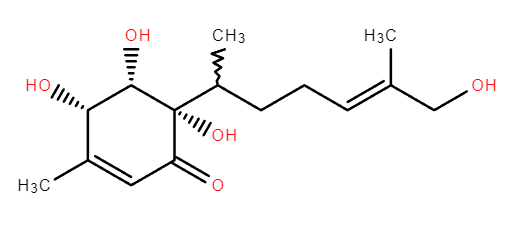 | _ | _ | _ |
|  | O20 | 3，4-Dihydroxybenzalacetone | C_10_H_10_O_3_ | 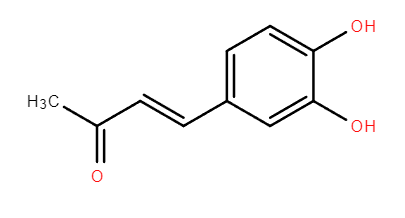 | _ | _ | _ |
|  | O21 | 1，2-benzenediol | C_6_H_6_O_2_ | 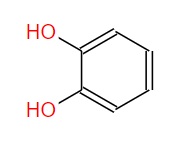 | 29.86 | 0.02 | MOL000089 |
|  | O22 | aspongopusin | C_10_H_9_NO_3_ | 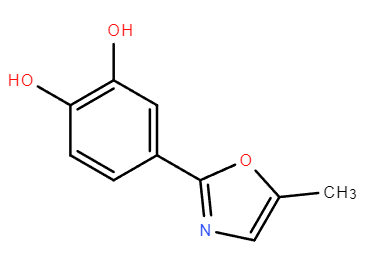 | _ | _ | _ |
|  | O23 | 3，4-dihydroxybenzene ethanol | C_8_H_10_O_3_ | 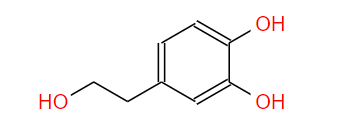 | 57.57 | 0.03 | MOL000141 |
|  | O24 | 3，4-dihydroxyphenylacetic acid | C_8_H_8_O_4_ | 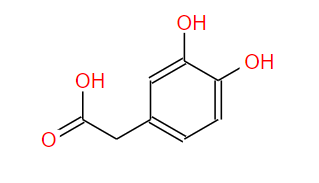 | _ | _ | _ |
| Other ingredients | O25 | vanillic acid | C_8_H_8_O_8_ | 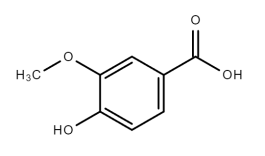 | 35.47 | 0.09 | MOL000114 |
|  | O26 | 4-hydroxyisobenzofuran-1( 3H) -one | C_8_H_6_O_3_ | 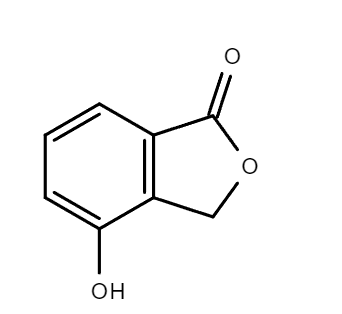 | 64.53 | 0.04 | MOL010576 |
|  | O27 | 2，4，5-trimethoxybenzaldehyde | C_10_H_12_O_4_ | 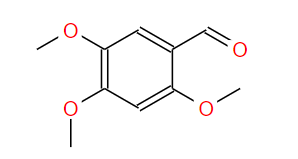 | 74.63 | 0.06 | MOL003554 |
|  | O28 | 2，3-butanediol | C_4_H_10_O_2_ | 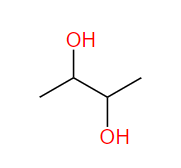 | 38.61 | 0.01 | MOL011087 |
|  | O29 | 1，2-propanediol | C_3_H_8_O_2_ | 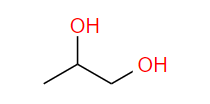 | _ | _ | _ |
|  | O30 | 2-hydroxy-5-( 2-hydroxyethyl ) phenyl β-D-glucopyran-oside | C_14_H_20_O_8_ | 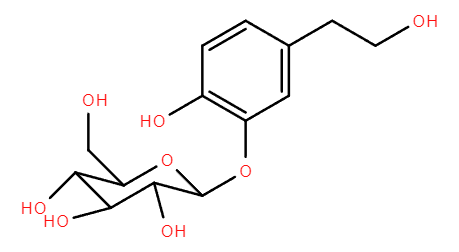 | _ | _ | _ |
|  | O31 | cyclo-( L-Leu-L-Trp) | C_17_H_21_N_3_O_2_ | 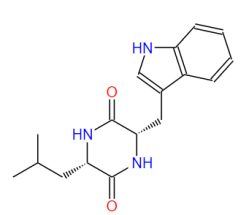 | _ | _ | _ |
|  | O32 | 2-hydroxy-3-methylbutanoic acid | C_5_H_10_O_3_ | 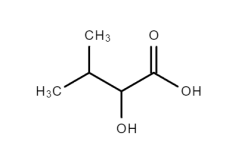 | _ | _ | _ |

NO: The classification and ordering of each component. OB: Oral bioavailability. DL: Drug-likeness. OB, DL and MOLID are from the TCMSP database. "-"means not application.
